# The microbiome and the gut-lung axis in tuberculosis: interplay in the course of disease and treatment

**DOI:** 10.3389/fmicb.2023.1237998

**Published:** 2023-10-31

**Authors:** Néstor Alvarado-Peña, David Galeana-Cadena, Itzel Alejandra Gómez-García, Xavier Soberón Mainero, Eugenia Silva-Herzog

**Affiliations:** ^1^Clínica de Tuberculosis, Instituto Nacional de Enfermedades Respiratorias “Ismael Cosío Villegas”, México City, Mexico; ^2^Laboratorio de Inmunobiología y Genética, Instituto Nacional de Enfermedades Respiratorias, México City, Mexico; ^3^Tecnológico de Monterrey, Escuela de Medicina y Ciencias de la Salud, México City, Mexico; ^4^Departamento de Ingeniería Celular y Biocatálisis, Instituto de Biotecnología, Universidad Nacional Autónoma de México, Cuernavaca, Mexico; ^5^Laboratorio de Vinculación Científica, Facultad de Medicina-Universidad Nacional Autonoma de México-Instituto Nacional de Medicina Genomica, México City, Mexico

**Keywords:** tuberculosis, *Mycobacterium tuberculosis* (MTB), gut-lung axis, microbiome, microbiota, anti-tuberculosis treatment

## Abstract

Tuberculosis is a chronic infectious disease caused by *Mycobacterium tuberculosis* (MTB) that remains a significant global health challenge. The extensive use of antibiotics in tuberculosis treatment, disrupts the delicate balance of the microbiota in various organs, including the gastrointestinal and respiratory systems. This gut-lung axis involves dynamic interactions among immune cells, microbiota, and signaling molecules from both organs. The alterations of the microbiome resulting from anti-TB treatment can significantly influence the course of tuberculosis, impacting aspects such as complete healing, reinfection, and relapse. This review aims to provide a comprehensive understanding of the gut-lung axis in the context of tuberculosis, with a specific focus on the impact of anti-TB treatment on the microbiome.

## Introduction

The human body contains a broad diversity of microorganisms, collectively known as the microbiota, which form a dynamic and functional system that evolves alongside its host. Although the gut harbors the largest population of microorganisms, they are also present throughout the body, including the entire digestive tract, skin, mucous membranes, urogenital and respiratory tract. This wide distribution underscores the significance of the microbiota in shaping and impacting various aspects of human health and physiology (Turnbaugh et al., 2007).

The millions of microbial cells in the human body play important roles in physicochemical and physiological functions, including intestinal development, barrier integrity and function, metabolism, immunity, inflammation, and neurological signaling regulation (Marsland et al., 2015; Enaud et al., 2020). The gut microbiome is highly dynamic and can be modified or disturbed by many factors, such as genetics, age, circadian rhythm, dietary habits, use of antibiotics, and other environmental factors (Nicholson et al., 2012; Dickson et al., 2016b). Furthermore, these factors play a role in the susceptibility, pathogenesis, and development of both non-transmissible and infectious diseases (Dang and Marsland, 2019; Naidoo et al., 2019; Wypych et al., 2019). In particular, malnutrition, diabetes, obesity, alcoholism, smoking, and HIV are some of the conditions that result in gut microbiome dysbiosis and altered immune function, that are associated with increased susceptibility to disease (Zevin et al., 2016; Weiss and Hennet, 2017; Iddrisu et al., 2021; Bach et al., 2023).

The increased intestinal permeability derived from this altered immune response and chronic inflammation allows metabolites and microorganisms to leak into the bloodstream, where they can affect other anatomical parts of the body, including the respiratory system (Usuda et al., 2021). Likewise, clinical studies on chronic lung diseases suggest that pulmonary disorders may be implicated in intestinal diseases (Rutten et al., 2014; Gui et al., 2021). Interestingly, the respiratory and gastrointestinal epithelia have structural similarities (Budden et al., 2017) and, in fact, several pulmonary and intestinal diseases exhibit many overlapping components, including common risk factors like mucus reduction, increased permeability, and low expression of tight-junction proteins, that can exacerbate the progression of infections (Duarte et al., 2018).

Tuberculosis (TB) is a chronic infectious disease caused by *Mycobacterium tuberculosis* (MTB) that persists as one of the top 13 causes of death worldwide (World Health Organization [WHO], 2022a). TB mainly affects pulmonary parenchyma presenting sustained weight loss, night sweats, fever, chronic cough, wasting, and hemoptysis. Diagnosis relies on identifying the microorganism through an automated PCR test (Xpert MTB/RIF and Xpert Ultra) (World Health Organization [WHO], 2021b). However, the heterogeneity of the TB clinical spectrum delays diagnosis and, therefore, anti-TB treatment (Cadena et al., 2017). Furthermore, anti-TB treatment represents one of the longest-duration antibiotic regimens used globally. This treatment includes combinations of at least four specific and broad-range antibiotics in schedules that range from four to more than 20 months, depending on the strain of MTB infection (World Health Organization [WHO], 2022b,c). Regardless of the regime, anti-TB treatment is associated with alterations of the gut microbiota in patients and animal models; the effect of these alterations in the lung microbiome and the underlying immune system response is the focus of many studies (Langdon et al., 2016; Namasivayam et al., 2017; Naidoo et al., 2019; Wang et al., 2020). This review aims to present a picture of recent studies on anti-TB treatment alterations of the microbiota in the course of the disease and its effect on the gut-lung axis.

## Gut-lung axis

The microbiome is a dynamic community of microorganisms that is in constant interaction with the host and its environment. Under physiological conditions, the microbiome is resilient to changes, benefiting both host and microbial communities, and it is considered to be in eubiosis (Giulio, 2021). On the other hand, the reduction of the adaptive capacity of a microbiome to changes that cause unfavorable alterations for the host is referred to as dysbiosis (Barbosa-Amezcua et al., 2022). All the different microbiomes in the human body: gut, lung, mouth, skin, genitals, liver and other barrier sites, are unique communities with specific interactions with the immune system and other organs in the body (Belkaid and Naik, 2013).

In particular, the host-associated gut microbiota is involved in several critical physiological functions such as absorption of nutrients, fermentation of food, vitamin production, and importantly, stimulating and training the immune system (Shreiner et al., 2015; Hillman et al., 2017; Al Nabhani et al., 2019). The gut microbiota includes bacteria, archaea, fungi, protozoa and viruses. Its composition is dominated by six bacterial phyla: Firmicutes, Bacteroidetes, Actinobacteria, Proteobacteria, Fusobacteria, and Verrucomicrobia, and two fungi phyla: Ascomycota and Basidiomycota (Nash et al., 2017). Although the composition changes with geographic location, diet, and age, it reaches a stable composition in absence of antibiotic treatment (Ferrer et al., 2017).

The interaction among all the organ systems is essential for the proper functioning of the body. Traditionally, this communication has been studied in the context of the autonomic nervous system, immune responses, and the endocrine system. However, recent research highlights a novel dimension of bidirectional communication between the gut microbiome and other organs such as the brain, skin, and lungs. These interactions constitute what is now recognized as the gut-brain axis, gut-skin axis, and gut-lung axis of microbiome communication, with each axis playing a significant role in maintaining overall health (Enaud et al., 2020; De Pessemier et al., 2021; Giulio, 2021). Despite the physical separation of the gut and lungs, microorganisms and immune cells communicate with each other resulting in immune tolerance to innocuous stimuli, host defense against potentially harmful external agents and pathogens as well as prevention of commensals from over-exploitation of host resources (Lazar et al., 2018; Yoo et al., 2020; Zheng et al., 2020).

**FIGURE 1:**
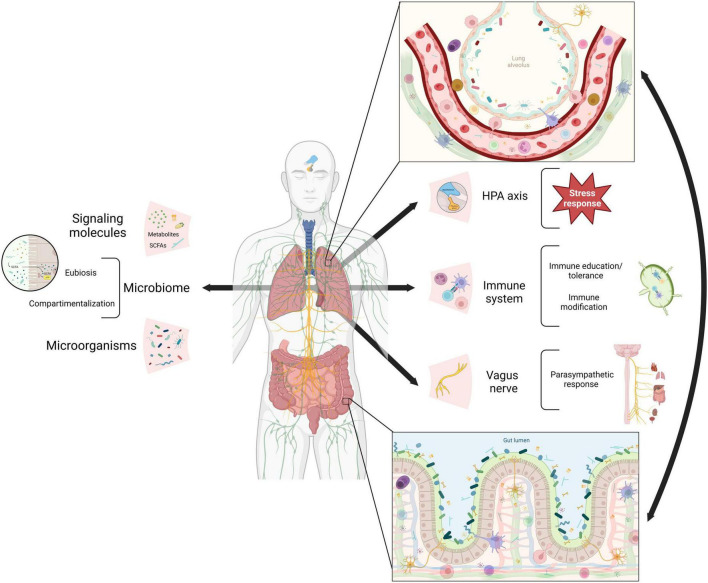
Mechanisms of gut-lung axis communication. Gut and lung communication pathways reported so far in literature (arrows). The neuroendocrine system via the hypothalamic-pituitary-adrenal (HPA) axis and the vagus nerve plus the immune system, cellular and soluble factors. All of them interact with microbiome signals from microorganisms and their metabolites. In both epitheliums, pathways’ components link up (zoom in) resulting in adequate stress and parasympathetic responses, immune education and modification, eubiosis and microorganisms compartmentalization (brackets). SCAFs (short-chain fatty acids). Created with BioRender.com.

Although the precise mechanisms of communication between the gut and lungs are not yet fully understood, emerging evidence points to the involvement of various pathways, including neuroendocrine and immune systems, as well as the translocation of microorganisms (Table 1 and Figure 1). These pathways often involve the release of metabolites, including microbiome-derived, that can shape immune responses, and modulate intestinal homeostasis and hematopoietic precursors in the bone marrow (Dang and Marsland, 2019). The vagus nerve, which connects the brain to multiple organs, including the lungs and gastrointestinal tract, is an essential conduit for this communication (Yuan and Silberstein, 2016). Onyszkiewicz et al. (2019) reported that butyric acid, a short-chain fatty acid (SCFA) produced by gut microbiota, lowers arterial blood pressure via colon-vagus nerve signaling. Furthermore, recent evidence has shown that the gut microbiota influences the hypothalamic-pituitary-adrenal (HPA) axis and the body’s response to stress (Frankiensztajn et al., 2020). In particular the intake of *Lactococcus lactis* was shown to lower the basal activity of the HPA axis, improve sleep, mental health and immune response through the activation of MQs and NK cells (Jin et al., 2020; Matsuura et al., 2022).

**TABLE 1:** Mechanisms of gut-lung axis communication.

Mechanism			Model	Key findings	Study
**Neuroendocrine**					
	Vagus nerve		mice	Vagal nerve stimulation prevents acute lung injury after trauma-hemorrhagic shock via the intestinal barrier protective effects provided by stimulation of the enteric nervous system.	Reys et al., 2013
	HPA axis		mice	*E. coli* and their LPS production can increase the occurrence of anxiety by inducing NF- kB activation.	Jang et al., 2018
**Immune response**					
	Immune education		mice	Early-life exposure to microbiota is important for the development of a normal and equilibrated immune system.	Al Nabhani et al., 2019
			mice	Innate lymphoid cells (ILCs) undergo maturation through the lung-gut axis to obtain proper function. A defect of ILCs development in the lung significantly impacts the count and function of ILCs in the gut.	Zhao et al., 2022
			mice	Comensal microbiota regulates generation of virus specific CD4 and CD8 T cells after influenza infection. Comensal microbiota leads to expression of IL-1beta, pro-IL18; activation of inflammasome.	Ichinohe et al., 2011
	Immune modification		mice	Commensal bacteria-derived ATP activates CD70 high CD11c low cells in the lamina propria to induce IL-6 and IL-23 production as well as TGF-b activation, thereby leading to local differentiation of T H 17 cell	Atarashi et al., 2008
			67 patients with asthma	Expression of TH 17-related genes was associated with Proteobacteria	Huang et al., 2015
**Signaling molecules**					
	**SCFAs**				
		Acetate	mice	Acetate-GPR43 interactions profoundly affect inflammatory responses. Stimulation of GPR43 by acetate was necessary for the normal resolution of colitis, arthritis and asthma	Maslowski et al., 2009
		Propionate	mice	Propionate on Ozone exposure induce airway hyperresponsiveness	Cho et al., 2018
		Butyrate	rat	Butyric acid lowers blood pressure via colon vagus	Onyszkiewicz et al., 2019
	**Tryptophan and derivatives**				
		Indole	mice	The microbiome metabolite indole reduced pulmonary and extrapulmonary bacterial burden, restored immune responses, and improved cellular trafficking required for host defense.	Samuelson et al., 2021
**Translocation of microorganisms**					
			mice and 68 patients with acute respiratory distress syndrome	Gut–lung translocation and alteration of the lung microbiome may represent a mechanism of pathogenesis in sepsis and ARD	Dickson et al., 2016b
			patient of intensive care unit (ICU)	Lung colonization in the ICU was driven by the translocation of *Pseudomonas aeruginosa* from the gut.	Wheatley et al., 2022

Summary of the main mechanisms associated with the communication in the gut-lung axis (Atarashi et al., 2008; Maslowski et al., 2009; Ichinohe et al., 2011; Cho and Blaser, 2012; Reys et al., 2013; Huang et al., 2015; Dickson et al., 2016a; Jang et al., 2018; Al Nabhani et al., 2019; Onyszkiewicz et al., 2019; Samuelson et al., 2021; Wheatley et al., 2022; Zhao et al., 2022).

The interaction between the gut microbiome and the respiratory system through the immune system is complex and dynamic; the microbiome exposes immune cells to a diverse range of antigens and microbial molecules, shaping its development and function, whereas the immune system maintains a permissive environment for the microbiota (Belkaid and Naik, 2013; Zheng et al., 2020). Both branches of the immune system participate in this communication. The innate immune system confers compartmentalization, preventing microbial translocation through a dense mucus layer, antimicrobial peptides (AMP), and tight junction proteins that preserve the epithelial barrier (Thaiss et al., 2016). Furthermore, the response of innate immune cells such as macrophages, dendritic cells, neutrophils, innate lymphoid cells, and epithelial cells respond to both commensal microbes signals and microbe-associated molecular patterns (MAMPs) (Chunxi et al., 2020). On the other hand, the gut commensal microbiome supports the production of secretory IgA by the adaptive immune system, which shapes microbial communities (Huus et al., 2021).

The integrity of the intestinal and lung epithelial barriers is crucial to prevent the translocation of microorganisms between the gut and respiratory tract and to maintain the internal physicochemical characteristics of both anatomical structures. However, the intestinal and lung epithelial barrier can be compromised under specific circumstances such as microaspirations, critical illness, sepsis, or chronic inflammation (Kang et al., 2023). As a result, microorganisms can translocate from the gut to the respiratory tract, potentially leading to the colonization of the respiratory tract by gut-derived microorganisms and contributing to the development or increase of severity of respiratory infections (Dickson et al., 2015; Wheatley et al., 2022). Similarly, respiratory system microorganisms, such as *Streptococcus pneumoniae*, have the potential to colonize the gastrointestinal tract (Floeystad et al., 2020). Furthermore, studies on a mice model, showed that the intratracheal inoculation of Lipopolysaccharides result in lung and gut microbiome perturbations with a parallel increase of bacterial load in the blood (Sze et al., 2014), underscoring the close interaction between these sites.

The dysbiosis and the resulting inflammation in one or both organs may contribute to the development of disease (Fabbrizzi et al., 2019). These interactions are influenced by immune cell migration and microbial metabolites in response to infection or inflammation (McGhee and Fujihashi, 2012; Zhao et al., 2022). Microbial metabolites produced by gut microbiota, such as SCFAs, tryptophan, secondary bile acids and their derivatives, modulate immune and epithelial cells (Agus et al., 2018; Ashique et al., 2022). SCFAs are a preferred energy source for colonocytes; they regulate the integrity of the intestinal barrier by inducing the secretion of IL-18 and antimicrobial peptides and the expression of the tight junctions. SCFAs inhibit macrophage production of proinflammatory cytokines and regulate T cell differentiation to Th1, Th17, and Tregs, thus are a central component of this interaction (Sun et al., 2017; Sencio et al., 2021).

Overall, the gut-lung axis is a complex and multifaceted system involving interactions between immune cells, microbiota, and signaling molecules from both systems. The response as a whole will depend on the health conditions and comorbidities of the individual and the different disease etiologies, which highlights the importance of understanding these interactions in different pathological conditions. An important factor to consider is the profound effect of antibiotics on the gut microbiome which have been found to have an increased risk for respiratory diseases in human studies as well as animal models (Ichinohe et al., 2011; Metsälä et al., 2015; Anand and Mande, 2018). The role of the gut-lung axis in tuberculosis has gained increasing recognition in recent years, highlighting its significance in the context of this infectious disease (Naidoo et al., 2019). Several studies have revealed that gut microbial dysbiosis can exacerbate lung inflammation and contribute to a dysregulated immune response to *M. tuberculosis* (Sekyere et al., 2020; Comberiati et al., 2021).

## Gut-lung axis and the impact of tuberculosis treatment

Tuberculosis is a disease that has co-evolved with humankind for millennia. Infection with MTB can result in a dynamic spectrum of clinical manifestations that range from elimination to asymptomatic latent TB to clinically active TB. Several factors influence these dynamic states, notably the immune response, microbiota, and the interaction between them. The main risk factors for tuberculosis include HIV infection [Relative Risk (RR) 18], alcohol use disorders (RR 3.3), undernourishment (RR 3.2), smoking (RR 1.6), and diabetes (RR 1.5) (World Health Organization [WHO], 2021a), all of which are associated with gut dysbiosis and proinflammatory susceptibility.

Of particular importance is the fact that MTB-infected individuals often have delayed diagnosis or undergo non-tuberculosis antibiotic treatment before a specific TB treatment is prescribed (Shi et al., 2021; Teo et al., 2021); in both instances, the resulting microbiome dysbiosis may increase the severity of the disease (Hogan et al., 2017). Broad-spectrum antibiotics, including cephalosporins and fluoroquinolones, are among the most frequently empirically prescribed antibiotics. Specifically, a decrease in the abundance of *Roseburia*, *Kluyvera*, and *Citrobacter genera*, and a near depletion of SCFA-producing bacteria, have been reported in these TB patients (Shi et al., 2021). Thus, gut microbiome dysbiosis, with a predisposition to inflammatory response, is expected in most patients secondary to the start of empirical antibiotic treatment, even before starting specific anti-TB treatment.

### Drug-susceptible MTB infection

Tuberculosis can be caused by MTB strains that are either resistant or susceptible to a variety of drugs. Between 2018 and 2021, 26.3 million TB patients were treated, of which 25.6 million were drug-susceptible (DS) and 649,000 drug-resistant (DR) (World Health Organization [WHO], 2022a). It is important to emphasize that treatments for tuberculosis are among the most prolonged antibiotic treatments approved by WHO; they range from four to 6 months for DS MTB and up to 20 months for DR MTB (World Health Organization [WHO], 2022b,c). These treatments include a combination of broad-spectrum and narrow-spectrum drugs with mycobacterial-specific targets (Table 2).

**TABLE 2:** Drug-resistant anti-TB treatment.

Groups and steps	Medicine	Abbreviation	Antibiotic spectrum	Dysbiosis time	Alteration in the microbiota	Model	References
Group A: Include all three medicines	Levofloxacin or moxifloxacin	Lfx Mfx	Broad	10 months	Decrease abundance of Alistipes, Bilophila, Butyricimonas, Coprobacillus, Faecalibacterium, Odoribacter, Oscillibacter, Parasutterella, Roseburia, Sutterella, Kluyvera, and Citrobacter genera.	Human	Dethlefsen et al., 2007; De Gunzburg et al., 2018; Burdet et al., 2019; Shi et al., 2021
	Bedaquiline	Bdq	Narrow	Unknow	Decrease *Streptococcus mutans.*	*In vitro*	Zhang et al., 2021
	Linezolid	Lzd	Broad	Unknow	Increase abundance of resistant *Enterococci* in the gut and an overall decrease of Gram-positive bacteria.	Human	Bourgeois-Nicolaos et al., 2014
Group B: Add one or both medicines	Clofazimine	Cfz	Narrow	Unknow	Unknow	Unknow	Unknow
	Cycloserine or terizidone	Cs Trd	Broad	Unknow	Decrease *abundance of Bifidobacterium* species and other butyrate producers.	Human	Minichino et al., 2021
Group C: Add to complete the regimen and when medicines from Groups A and B cannot be used	Ethambutol	E	Narrow	Unknow	Unknow	Unknow	Unknow
	Delamanid	Dlm	Narrow	Unknow	Unknow	Unknow	Unknow
	Pyrazinamide	Z	Narrow	Unknow	Decrease abundance of *Clostridia species* and increase *Anaeroplasma*.	Murine	Namasivayam et al., 2017
	Imipenem- cilastatin	Ipm-Cln	Broad	Unknow	Decrease abundance of *Enterobacteria*, *Enterococci*, *Bifidobacteria*, *Eubacteria*, *Lactobacilli*, and *Bacteroides*.	Human	Bhalodi et al., 2019
	Meropenem	Mpm	Broad	Unknow	Decrease abundance of *Enterobacteria*, *Clostridia*, and *Bacteroides* and increase *Enterococci.*	Human	Bhalodi et al., 2019
	Amikacin (or streptomycin)	Am (S)	Broad	Unknow	Decrease abundance of *Bacteroidales*, *Clostridiales* and increases in the *Lachnospiraceae* and *Bacteroidaceae*.	Murine	Lichtman et al., 2016
	Ethionamide or prothionamide	Eto Pto	Narrow	Unknow	Unknow	Unknow	Unknow
	P-aminosalicylic acid	PAS	Narrow	Unknow	Unknow	Unknow	Unknow

Principal regimen options for drug-resistant tuberculosis (Dethlefsen et al., 2007; Bourgeois-Nicolaos et al., 2014; Lichtman et al., 2016; De Gunzburg et al., 2018; Bhalodi et al., 2019; Burdet et al., 2019; Minichino et al., 2021; Shi et al., 2021; Zhang et al., 2021; World Health Organization [WHO], 2022c).

The WHO standard recommended scheme for DS MTB consists of four essential drugs designated as “first-line” anti-TB treatment: isoniazid (H), rifampicin (R), pyrazinamide (Z), and ethambutol (E) for 2 months, followed by 4 months of only HR; recently the WHO added moxifloxacin (Mfx) and rifapentine (Rpt, a synthetic derivative of rifampicin) to primary treatment. Rifampicin, and moxifloxacin are broad-spectrum antibiotics used in other non-mycobacterial infections, whereas isoniazid, pyrazinamide and ethambutol have mycobacterial-specific targets. Two alternative DS treatments have been recently approved by WHO; one includes a 2-month treatment of Rpt, moxifloxacin (Mfx), H and Z followed by 2 months with RptHMfx (Dorman et al., 2021; World Health Organization [WHO], 2022c), and the second one, a 2-month treatment of bedaquiline (Bdq), Linezolid (Lzd) and HZE, which recently proved their effectiveness in clinical trials (Paton et al., 2023). Both of these new alternative treatments significantly decrease the time of treatment but contain broad-spectrum antibiotics (Lzd and Mfx) that result in broader damage to gut microbiota and should be evaluated accordingly (Dorman et al., 2021; Paton et al., 2023).

The effect of each of these antibiotics in the microbiome cannot be evaluated individually on tuberculosis patients. However, several studies of broad-spectrum antibiotics used in anti-TB treatments on healthy individuals have shown drastic and long lasting effects in the gut microbiome. For example, 5 days treatment of ciprofloxacin or Mfx resulted in a drastic reduction in alpha diversity, characterized by a decreased abundance in *Alistipes*, *Bilophila*, *Butyricimonas*, *Coprobacillus*, *Faecalibacterium*, *Odoribacter*, *Oscillibacter*, *Parasutterella*, *Roseburia*, and *Sutterella* genera (De Gunzburg et al., 2018; Burdet et al., 2019). Similarly, studies with Lzd showed an increase of resistant *Enterococci* in the gut and an overall decrease of Gram-positive bacteria cells in the nasal, pharyngeal, and intestinal microbiomes (Bourgeois-Nicolaos et al., 2014). Furthermore, antibiotic therapies of first-line anti-TB medications (R or HZ) in murine models demonstrated changes in taxonomic composition and a decreased alpha and beta diversity; Rifampicin lead to an expansion of *Bacteroides*, *Verrucomicrobiaceae*, and a decrease in *Lachnospiraceae* families. Unexpectedly, the treatment with HZ, mycobacterial specific drugs, resulted in an expansion, although a modest one, of Bacteroidetes, particularly the *Clostridiaceae* family (Khan et al., 2019).

**FIGURE 2:**
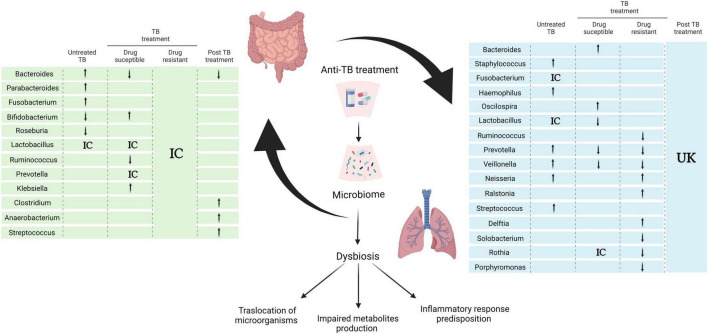
Effect of anti-TB treatment on the gut- axis microbiome communication. Alterations of gut and lung microbiota in the course of anti- TB treatment according to selected references using research words “TB treatment,” “microbiome,” “tuberculosis” “microbiota” in PubMed database NCBI. Arrows indicate genera enrichment or decreased, IC (inconclusive) denotes controversial or non-significant results within references and unknown (UK) and blank spaces for missing data in the references reviewed (Botero et al., 2014; Khan et al., 2016; Krishna et al., 2016; Namasivayam et al., 2017; Wipperman et al., 2017; Vázquez-Pérez et al., 2020; Wang et al., 2020; Cao et al., 2021; Kateete et al., 2021; Lin et al., 2021; Valdez-Palomares et al., 2021; Xiao et al., 2022; Ye et al., 2022; Zhang et al., 2022). Created with BioRender.com.

The consequence of initial TB treatment on the microbiome has implications for the overall outcome: relapse, reinfection, and perhaps the severity of the disease (Khan et al., 2016; Hu et al., 2019). Thus it is important to understand its implications in the development of disease as well as in the patient’s overall state of health. Several studies have shown a gut microbiome dysbiosis during first-line anti-TB treatment for DS TB that encompasses both bacteria and fungi. A decrease in abundance of the bacterial genera *Ruminococcus*, *Eubacterium*, *Lactobacillus*, *Coprococcus*, *Dialister*, *Dorea*, *Bacteroides*, and *Oscillospirales*, and simultaneous increase of *Erysipelatoclostridium*, *Veillonella*, *Bifidobacterium*, *Klebsiella*, and *Prevotella* have been reported (Wipperman et al., 2017; Meng et al., 2022; Figure 2). Whereas, an increase in the relative abundance of the fungi genera *Purpureocillium*, *Nakaseomyces*, *Rhodotorula*, and *Genolevuria*, with a decrease in *Naganishia* and *Mucor* genera (Cao et al., 2021) was shown.

This dysbiosis results in an overall decrease of microbial SCFAs production, which has been associated with a weakened intestinal epithelial barrier, reduction of mucin and AMP expression with the corresponding exacerbation of systemic inflammatory response (Sun et al., 2017; Zhang et al., 2023). Although studies of respiratory tract microbiome are fewer and harder to compare due to differences in sample and study design, they do confirm disruption of the microbiome affected by MTB infection and treatment; overall an increase abundance of *Bacteroides* and *Oscillospira* and a decrease in *Lactobacillus*, *Prevotella*, and *Veillonella* has been reported (Figure 2; Valdez-Palomares et al., 2021; Zhang et al., 2022).

Furthermore, when oral antibiotics cannot be used, patients may require intravenous antibiotics like carbapenems. However, carbapenems for anti-TB treatment are prescribed in conjunction with clavulanic acid, since MTB has a constitutive beta-lactamase BlaC, that has a penicillinase, cephalosporinase, and carbapenemase activity that is inhibited by clavulanic acid (Bhattacharya et al., 2021). Moreover, in México and other Latin American countries, clavulanic acid is administered with amoxicillin, which adds another broad-spectrum antibiotic to the treatment (National Center for Preventive Programs and Disease Control, 2020). The administration of these antibiotics results in further changes in the gut microbiota, including the increase of the *Bacteroidales* order and *Bifidobacterium* species in the gut microbiota (Gaucher et al., 2021). Even monotherapies of carbapenems have shown drastic effects on the gut microbiota. In particular, meropenem administration in healthy volunteers decreased the abundance of *Enterobacteria*, *Clostridia*, and *Bacteroides* and increased *Enterococci*, while genera like *Bifidobacterium* and *Lactobacillus* remain stable. On the other hand, imipenem was shown to reduce all of the species mentioned, with only *Clostridia* remaining stable (Bhalodi et al., 2019).

### Drug-resistant MTB infection

Although drug-resistant tuberculosis (DR-TB) corresponds to only 4.2% of total MTB infections in 2021, it has steadily increased in recent decades, from 30,000 cases in 2009 to 450,000 in 2021 (World Health Organization [WHO], 2022a). DR-TB has been divided by the WHO into five categories: rifampicin-resistant (RR), isoniazid-resistant, and rifampicin susceptible (Hr), multidrug-resistant (MDR), defined as H and R resistant; pre-extensively drug-resistant (pre-XDR-TB) which refers to TB that is resistant to R (may also be resistant to H), and any fluoroquinolone; whereas extreme drug-resistant TB (XDR-TB), is resistant to R, (may also be resistant to H), any fluoroquinolone, plus at least one of either Bdq or Lzd (World Health Organization [WHO], 2022b).

Currently, treatment of drug-resistant infection is individualized and includes broad-spectrum as well as mycobacterial-specific antibiotics (see Table 2). In 2022, the WHO renewed its recommendations for DR treatment to include three drugs from Group A and at least one from Group B or Group C, depending on the susceptibility pattern and the location of the infection (World Health Organization [WHO], 2022b). Furthermore, newer shorter schemes that include BPaL (Bedaquiline, Pretomanid, Linezolid), or BPaLM (BPaL + Moxifloxacin) for 6 months are being introduced (World Health Organization [WHO], 2022b).

Similar to DS treatment, DR-TB treatment leads to profound changes on the gut microbiome and, thus, impacts the gut-lung axis. Alterations in the gut microbiota of DR-TB-treated patients have been reported in terms of overall decrease in alpha diversity that can last for years after treatment completion (Wang et al., 2020; Shi et al., 2021). In particular, an increase of *Enterobacteriaceae* is seen from healthy to RR and MDR, along with a decrease in members of the phylum Actinobacteria and Firmicutes in MDR patients (Wang et al., 2020; Shi et al., 2022). Furthermore, phylum Verrucomicrobia was found as a predominant component in Pre-XDR-TB, whereas it is almost undetectable in healthy, RR or MDR individuals (Shi et al., 2022). On the other hand, studies on the macaque model have shown an increase in Proteobacteria in RR and MDR but not in Pre-XDR-TB or healthy controls (Namasivayam et al., 2019). Moreover, members of the Bacteroidetes phylum were only found in healthy individuals. Gut-derived metabolites, such as SCFAs, tryptophan and secondary bile acids, decreased from MDR to Pre-XDR and RR to healthy participants, underscoring a complex interaction between the microbiota and immune system (Shi et al., 2022). Studies of monotherapies, although not in TB patients, particularly cycloserine treatment, a group B drug, reduces *Bifidobacterium* species and other butyrate producers in the gut microbiota (Minichino et al., 2021). Overall there are clear changes in the composition and diversity of the microbiota, but inconsistent in terms of specific taxa abundance (Table 2).

The latest treatments of TB include the new anti-TB drugs: bedaquiline, delamanid, and pretomanid; the first new anti-TB drugs to be approved in 40 years. Bdq and Dlm/Pto target mycobacterial respiratory chain components, including the ATP-synthase. These drugs are recommended for some forms of RR, MDR, or Pre-XDR and XDR (World Health Organization [WHO], 2022b). Although there is limited information on the effect of either of these drugs on the microbiome recent research showed an inhibition of proliferation and biofilm production of *Streptococcus mutans*, and other oral pathogens after Bdq treatment, which stresses the impact of this antibiotic on the microbiome in general, not only to MTB (Zhang et al., 2021).

### Long-term effect of anti-TB treatment on the gut-lung axis

The gut microbiota dysbiosis, consequence of any antibiotic treatment, results in an altered immune response and increased vulnerability to other infections. There is a reduction in the expression and secretion of AMPs, including C-type lectins, defensins, and cathelicidins; compromised integrity of the epithelial barrier, as well as reduced production of SCFAs, all of which are part of the first line of defense to incoming pathogens (Schumann et al., 2005; Hill et al., 2010; Willing et al., 2011; Wipperman et al., 2017). Common and recurrent *Clostridium difficile* infections, as well as increased susceptibility to *Salmonella enterica* and *Escherichia coli* infections after antibiotic exposure, have been reported (Croswell et al., 2009; Wang et al., 2020). Furthermore, reduced butyrate has been associated with neutrophil infiltration and T cell anergy (Meijer et al., 2010). Thus it is possible that anti-TB treatment has the side effect of hampering the immune response against the mycobacteria.

After completion of antibiotic therapy, the dysbiotic microbiome will either return to the initial state before treatment or establish a new eubiosis. This process involves cooperation and competition among the microorganisms as well as the changes in the physicochemical properties of the gut tract, which is affected by the length of the treatment and the type of drugs involved. For example, the dysbiosis caused by a 5-day fluoroquinolone treatment is reversed after a 4-week recovery period (Dethlefsen et al., 2007). However, a 6-month DS treatment results in a dysbiosis that lasts at least 1.2 years, and a 20-month MDR treatment may have irreversible consequences for the microbiome (Wipperman et al., 2017; Wang et al., 2020). Furthermore, during anti-TB treatment, some bacteria enter dormancy or a persister state as a result of stressors, including hypoxia. It is possible that disease relapse, result of the activation of these persister bacilli, and increased susceptibility to reinfection is caused by diminished immune control consequence of gut-lung microbiome dysbiosis (Zhang et al., 2012; Quigley and Lewis, 2022).

The intricate relationship between antibiotic treatment, gut-lung microbiome dysbiosis, and tuberculosis outcomes make it evident that it is necessary to consider the microbiome as part of the treatment. For this, it is crucial to understand the impact of different treatments on the microbiome and its potential consequences for disease development. A promising new approach: “Host-directed-therapy” (HDT) aims to improve innate immunity, instead of targeting the pathogen directly. HDT has been used in antitumor therapies, inflammatory bowel disease and infectious diseases, is particularly important in the context of antibiotic resistance (Wei et al., 2015; Langdon et al., 2016; Bergman et al., 2020; Bustamante et al., 2020; Davar et al., 2021; He et al., 2021; Jeong et al., 2023). HDTs include the use of probiotics, prebiotics, symbiotics, microbiota transplants and phage therapy. HDT induces the activation of the endogenous defense mechanisms including antimicrobial peptides, reactive oxygen species, autophagy etc (Bergman et al., 2020; Diallo et al., 2021). For example, a clinical trial in Bangladesh, (Mily et al., 2015), showed improved MTB clearance after use of adjunct therapy of phenylbutyrate (a SCFA) and vitamin D3 in a standard short-course first line TB treatment. Adjunct therapy of Butyrate in Shigellosis also showed early reduction of local inflammation (Raqib et al., 2012). Furthermore, studies suggest that certain probiotic strains of *Lactobacillus* and *Lacticaseibacillus*, may have immunomodulatory effects and could enhance the body’s defense mechanisms against infections, including TB (Jiang et al., 2022; Rahim et al., 2022). Probiotics may help regulate inflammation, promote tissue repair, and a better immune response, all of which are important for patients with TB and post-TB recovery.

In conclusion, the gut microbiome cross talk with the immune response occurs and has an impact in the development of tuberculosis. Furthermore, therapeutic strategies that utilize gut microbiota and their metabolites in combination with the appropriate antibiotic treatment, may provide improved outcomes for patients.

## Discussion

There has been a great deal of research on *M. tuberculosis’s* long and complex interaction with its host. Many of the factors that contribute to the susceptibility and development of the disease are associated directly or indirectly with immune maintenance, including HIV infection, malnutrition, diabetes, smoking, and substance abuse. All of these conditions result in gut microbiome dysbiosis. In turn, gut dysbiosis has been implicated in disease development locally or distal, including in the respiratory tract. Although we are just beginning to understand the crosstalk in the gut-lung axis that allows passage of microbial and host metabolites, it has become clear that these interactions affect the susceptibility and development of many respiratory diseases, including tuberculosis.

Gut microbiota is altered from the initial lung infection of MTB and increases substantially with the long anti-TB treatments. TB treatment is one of the world’s most widely administered antibiotic combinations. The long-term effect of antibiotic treatments is evident; from 6 months of DS TB, treatment that can last up to a year, to potentially irreversible changes after a 20-month DR-TB treatment (Wang et al., 2020). The loss of bacterial diversity as a result of antibiotic treatment can lead to an increased vulnerability to infections, as has been shown for *C. difficile*, *E. coli*, and *S. enterica* (Croswell et al., 2009; Wang et al., 2020), and may be part of the explanation for high relapse or reinfection rates on DR-TB patients. Additionally, even with treatment adherence, 14% of DS and nearly 40% of DR TB patients fail treatment, and 5% of all patients with successful treatment relapse (Getahun et al., 2011; World Health Organization [WHO], 2022a). This suggests that the cure and prevention of relapse in tuberculosis may not depend solely on anti-TB treatment. The respiratory and gut microbiota dysbiosis and its interplay with the immune response play an important part. There is numerous evidence that demonstrates changes in the taxonomic composition as well as the overall diversity of the gut and respiratory microbiome during anti-TB treatment. However, probably due to differences in study design and samples taken, or individual characteristics of each patient, there are inconsistent results in terms of changes of specific organisms. To fully understand the interplay between the microbiome and host defense mechanisms, longitudinal studies that follow patients’ respiratory and gut microbiome through their treatments, integrating the immune response, are needed. Furthermore, we need to go beyond the study of only bacteria and include all other microorganisms in the microbiota as well as metabolome and resistome.

Although we have pointed out some of the adverse effects of antibiotic therapies, it is clear that antibiotic therapy for TB and other infectious diseases is a central tool for their treatment. However, strategies that reduce dysbiosis and restore a healthy microbial balance are needed. In tuberculosis management, current efforts include shortened and narrow spectrum antibiotic therapies, together with host-directed-therapies that improve immune response. There are promising results in the use of pre- and probiotic adjunct therapies in TB treatment; however, more clinical studies are needed to establish their effectiveness in this specific context. Patients’ individual characteristics, choice of pre or probiotics, dosages, timing need careful consideration.

In sum, the treatment of tuberculosis has broad public health implications, with millions of people being treated with first-line anti-TB medicines for 6 months, resulting in microbiome dysbiosis lasting years after treatment completion (Wipperman et al., 2017). Future research should aim to develop strategies that optimize treatment outcomes by considering the dynamic interplay between the microbiome and host immune responses.

## Author contributions

ES-H and NA-P: conceptualization. DG-C, IG-G, and NA-P: methodology. ES-H, DG-C, XM, IG-G, and NA-P: formal analysis, investigation, review and editing, and writing-original draft preparation. IG-G: visualization. ES-H: supervision and funding acquisition. All the authors contributed equally to writing and editing of the document, read, and agreed to the published version of the manuscript.
